# CRISPR/Cas9-Induced Loss of Keap1 Enhances Anti-oxidation in Rat Adipose-Derived Mesenchymal Stem Cells

**DOI:** 10.3389/fneur.2019.01311

**Published:** 2020-02-18

**Authors:** Yiling Hu, Shubao Liu, Bing-Mei Zhu

**Affiliations:** Regenerative Medicine Research Center, West China Hospital, Sichuan University, Chengdu, China

**Keywords:** gene editing, CRISPR/Cas9, traumatic brain injury, Keap1-Nrf2, anti-oxidation

## Abstract

Stem cells have become a powerful tool in the treatment of many diseases owing to their regenerative ability and rapid promotion of development in regenerative medicine such as in traumatic brain injury. However, the high level of oxidant micro-environment in lesion region leads to more than 99% cells into death. In this study, we used genetic methods to edit *Keap1* gene in mesenchymal stem cells, we and observed their antioxidative ability. First, we disturbed the start codon and the 376th amino acid codon of *Keap1* in adipose-derived mesenchymal stem cells (Ad-MSCs) with CRISPR/Cas9, respectively, to release Nrf2 from the binding of Keap1. As a result, Nrf2 was activated and localized into nuclei and regulated cellular anti-oxidation. We observed that the cells lacking *Keap1* ATG codon showed obvious nuclear localization of Nrf2. Besides lower expression of *Bax-1* and lower content of malondialdehyde (MDA) were detected after H_2_O_2_ treatment, we also found higher expression of *Bcl-2* in *Keap1* ATG codon knock-out cells, whereas a higher expression of *PCNA* was observed only in the *Keap1* 376th codon-edited cells, whose *Bax-1* expression was lower than that in the control cells. Our study revealed that loss of *Keap1* resulted in anti-oxidative ability in Ad-MSCs, suggesting that our strategy can hopefully increase the viability of mesenchymal stem cells after grafting. This study is also a frontier exploration to the application of CRISPR/Cas9 in Ad-MSCs.

## Introduction

Many neural degenerative diseases, like Alzheimer's diseases, Parkinson's diseases, stroke, epilepsy, and spinal cord injury, have become the greatest threats to the modern public health. Stem cell therapy is considered as an effective approach to repair tissue injury and treat these diseases (1–5). However, the low viability of stem cells after grafting impedes the progress of stem cell therapy. Studies have shown that more than 99% cells died at 4–7 days in the post-grafting (6), because cells from injured tissue produce a large amount of reactive oxygen species (ROS) in hypoxic–ischemic environment, and the accumulation of ROS impairs stem cell survival after grafting (7, 8). Beyond that, the inflammatory reactions induced by complex, variable, and hostile micro-environment in lesion areas become the culprits that torture the post-grafting stem cells to death. Therefore, it is important to enhance the anti-oxidation and anti-inflammatory property after traumatic brain injury. This effort may ensure higher success rate and therapeutic effect of mesenchymal stem cell grafting.

Nrf2, also known as nuclear factor erythroid 2-like 2 (NFE2L2), acts as a critical transcription factor in cells and regulates hundreds of downstream genes, which encode proteins related to anti-oxidation, such as glutamate-cysteine ligase catalytic and modifier subunits, glutathione peroxidase, glutathione reductase, thioredoxin, peroxiredoxin 1, and superoxide dismutase 3. The activation of Nrf2 reduces NF-κB expression in the cells and inactivates a series of inflammatory mediators, like cytokines, chemokines, adhesion molecules, COX-2, MMP-9, and iNOS (9). For these reasons, Nrf2 stands at the vital position in terms of cellular anti-oxidation and anti-inflammation. In physiological condition, kelch-like ECH-associated protein 1 (Keap1) interacts with Nrf2 in cytoplasm, induces ubiquitination and subsequent proteasome mediated degradation, and then results in the failure of Nrf2 nuclear localization (10). Thus, its existence inhibits Nrf2 from facilitating anti-oxidation and anti-inflammation of stem cells. Conversely, it seems feasible to increase the anti-oxidation and anti-inflammation of stem cells by disturbing the interaction between Keap1 and Nrf2 and promoting the Nrf2 nuclear localization (11, 12).

The CRISPR/Cas9 was found originally from the adaptive immune system of *Streptococcus pyogenes*, which uses a non-condoning RNA to guide a nuclease Cas9 and introduce double-stranded breaks (DSBs) on the invasive exogenous DNA targets, like bacteriophages (13). Thus, there are two components in CRISPR/Cas9 system, the nuclease Cas9 and the non-condoning RNA also called single-guide RNA (sgRNA). Each sgRNA is about 100-nt length, and for the first 20 nt of its front end, it works as a probe binding with the target sequence by complementary base pairing. For the other 80 nt of sgRNA, it works as a scaffold binding Cas9. Besides, that exogenous DNA target must contain a 5′-NGG protospacer adjacent motif (PAM), which can be recognized by Cas9. After the formation of DSBs, two repair mechanisms are activated: non-homologous end-joining (NHEJ) and homologous recombination. The former acts as a dominating DNA repair method when there is no donor DNA template existing, and as a result, insertions and/or deletions (INDELs) are created at the cut site of the target DNA sequence. The expression of the target gene will be disturbed with the formation of frameshift mutation that is caused by INDELs.

## Materials and Methods

### Stem Cell Produced

The stem cell was derived from rat adipose tissue, which is isolated and minced using a blade, and then digested with 0.1% collagenase type I (Gibco) for 2–3 h at 37°C oscillatingly. The digestion is terminated by isopyknic Dulbecco's modified Eagle's medium (DMEM) supplemented with 10% fetal bovine serum. The solution is then passed through a 100-μm filter and centrifuged at 1,400 rpm for 5 min, and then the supernatant is discarded, and the deposit is washed with DMEM twice. The adipose-derived mesenchymal stem cells (Ad-MSCs) are resuspended and cultured in a T75 flask with DMEM containing 10% fetal bovine serum and 1% penicillin/streptomycin (5).

### Single-Guide RNA Designs and Plasmid Constructs

**Figure 1:**
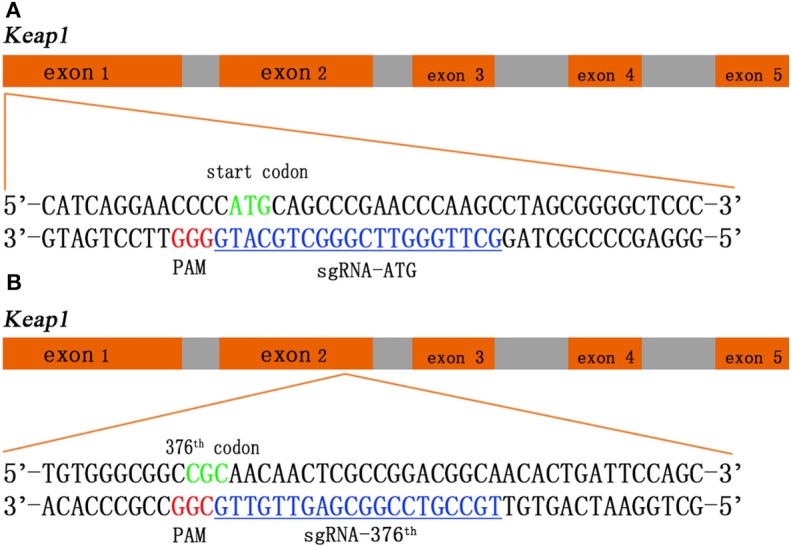
Schematic of the *Keap1* gene and the CRISPR/Cas9 guide RNA (gRNA) target site. Green sequences represent start codon **(A)** and 376th codon **(B)** of *Keap1*, red sequences represent protospacer adjacent motif (PAM), and blue sequences with underscore represent single-guide RNA (sgRNA).

Two sgRNAs were designed to target amino acid positions start codon and the 376th codon of *Keap1* sequence (Figure 1). To anneal the complimentary oligos, 8 μl of sense oligo + 8 μl of antisense oligo (both at 10 μM) + 2 μl of 10 × ligase buffer (NEB) were mixed, followed by melting and reannealing in a thermal cycler with the following program: 96°C for 300 s, followed by 85°C for 20 s, 75°C for 20 s, 65°C for 20 s, 55°C for 20 s, 45°C for 20 s, 35°C for 20 s, and 25°C for 20 s with a −0.3°C/s rate between steps. To phosphorylate the overhangs, 1 μl of 25 mM ATP + 1 μl of T4 Polynucleotide Kinase (NEB) were added and incubated at 37°C for 60 min followed by 65°C for 20 min to heat inactivate the enzyme. Then the CRISPR/Cas9 expression plasmid pU6-(*Bbs*I)_CBh-Cas9-T2A-mCherry (Addgene #64324) was digested with restriction enzymes *Bbs*I (Fisher Scientific) according to manufacturer's instructions. The DNA oligos of sgRNAs with sticky ends were ligated to the linearized CRISPR/Cas9 expression plasmid by T4 DNA ligase (NEB). The recombination plasmid was expanded with competent cell DH5-α and extracted with QIAGEN Plasmid Midi Kit (QIAGEN) according to manufacturer's instructions (14).

### Transfections and Cell Sorting

Rat Ad-MSCs were cultured with trypsin enzyme-digesting technique and passaged. The recombination CRISPR/Cas9 plasmids were electroporated into passage 2 Ad-MSCs by Lonza Nucleofector 4D as plasmids (5 μg) and Ad-MSCs (1 × 10^6^). Seventy-two hours later, transfected cells were sorted by fluorescence-activated cell sorting (FACS).

### DNA Extraction and Sequencing

Cells were cultured in 6-well-plates, and the genomic DNA was isolated using the Agencourt DNAdvance Genomic DNA Isolation Kit (Tiangen) according to the manufacturer's instructions. On-target genomic regions of interest were amplified by PrimeSTAR HS DNA Polymerase (Takara) and primers (Table 1). The PCR products were used to perform flanking high-throughput sequencing (Illumina NovaSeq6000).

**Table 1:** Primers of the target-site PCR for high-throughput sequencing.

**Target site**	**Primer sequences**	
sgRNA-ATG	Sense	5′-CAGCCCTGTACTCTTCCCTCCTT-3′
	Antisense	5′-TGCATACATCACCGCGTCCC-3′
sgRNA-376th	Sense	5′-TTACTTCCGACAGTCGCTCAG-3′
	Antisense	5′-CACGCTCATAGAGGCACAGG-3′

*sgRNA, single-guide RNA*.

### Western Blot

Total proteins were extracted with TRIzol (Invitrogen) from each group. After detection of protein concentration with BCA Kit (Thermo Fisher) according to the manufacturer's instructions, we performed the western blotting experiment. Samples were electrophoresed on sodium dodecyl sulfate–polyacrylamide gel electrophoresis (SDS-PAGE) with 60 V, 30 min and 120 V, 60 min. After being transferred to the polyvinylidene difluoride (PVDF) membrane with 300 mA for 150 min, the PVDF membrane was blocked in Tris-buffered saline (TBS)−0.05% Tween 20 with 5% non-fat milk in 37°C for 60 min, and then primary antibodies were incubated with 4°C overnight. On the second day, the PVDF membrane was incubated with secondary antibody after extensive wash, and then the protein bands were visualized by FUSION FX7 (VILBER).

### Immunofluorescence

Cells were seeded on glass-bottom 12-well-plates and fixed with 4% paraformaldehyde containing 0.1% Triton X-100 for 20 min at room temperature. Cells were washed three times with 1 × phosphate-buffered saline (PBS) and incubated with 1% bovine serum albumin (BSA) for 30 min at room temperature. Samples were incubated with anti-Nrf2 antibody (1:200 dilution) in the blocking buffer overnight at 4°C followed by the incubation with secondary antibody (1:1,000 dilution) for 2 h at room temperature. Images were collected by fluorescence microscopy (15).

### Oxidative Cell Damage and Measurement of Malondialdehyde

Cells were seeded in 6-well-plates in the media containing 200 μM of H_2_O_2_. Four hours later, cells were washed with 4°C PBS and lysed with cell lysis buffer for immunoprecipitation (IP) (Beyotime). Total protein was assayed with bicinchoninic acid (BCA) kit (Thermo Fisher) by following the provided instruction. Malondialdehyde (MDA) accumulation in the cells was measured using Lipid Peroxidation MDA Assay Kit (Beyotime) according to the manufacturer's instructions. The data were normalized by cellular protein concentrations.

### RT-qPCR Analyses

Total RNA was extracted from cells using TRIzol (Invitrogen) (16). cDNA synthesis was performed by PrimeScript™ RT reagent Kit with gDNA Eraser (Takara). RT-qPCR was performed using primers (Table 2) and TB Green™Premix Ex Taq™II (Takara) in a QuantStudio™ 5 RT-qPCR System (Applied Biosystems). All of operations above were followed according to the manufacturer's instructions. Expression levels were determined by a standard ΔΔCt method. β*-Actin* was analyzed as an internal control.

**Table 2:** Primers of gene *Bax-1, Bcl-2 PCNA*, and β-Actin for RT-qPCR.

**Gene name**	**Primer sequences**	
*Bax-1*	Sense	5′-GCAAACTGGTGCTCAAGGC-3′
	Antisense	5′-GGGTCCCGAAGTAGGAAAGG-3′
*Bcl-2*	Sense	5′-GGGAGCGTCAACAGGGAGAT-3′
	Antisense	5′-AGCCAGGAGAAATCAAACAGAGGT-3′
*PCNA*	Sense	5′-AAGGGCTGAAGATAATGCTGATAC-3′
	Antisense	5′-GTTCTGGGATTCCAAGTTGCT-3′
*β-Actin*	Sense	5′-CCCGCGAGTACAACCTTCTT-3′
	Antisense	5′-CGCAGCGATATCGTCATCCA-3′

### Statistical Analysis

Data are presented as mean ± SD and are compared by a paired, two-tailed Student's *t*-test for a single comparison between two groups. Statistical significance of difference was defined as a *p* ≤ 0.05.

## Results

We employ CRISPR/Cas9 system into rat Ad-MSCs to interrupt *Keap1* gene so that Nrf2 can be released from ubiquitination and proteolytic degradation and works in the cellular nuclei. We designed two sgRNAs in *Keap1* gene sequence targeting the start codon and the 376th codon arginine, which is a critical amino acid between interaction of Keap1 and Nrf2 by salt bridge, respectively (Figures 1A,B). With this tool, we could achieve the modification of *Keap1* expression in rat Ad-MSCs and lead to a controlled *Keap1* knock-out, therefore enhancing the ability of anti-oxidation and anti-inflammation.

We electroporated the recombinant CRISPR/Cas9 plasmids into passage 2 rat Ad-MSCs. Seventy-two hours later, we used FACS to collect mCherry positive cells that contain the CRISPR/Cas9 plasmids and expanded them for detection. Genomic DNAs were extracted, and the targeted DNA sequences were amplified by designed primers (Table 1). The PCR products were sequenced by high-throughput DNA sequencer. According to the results, we found that about 53% DNA sequence nearby ATG codon was disturbed (Table 3), and about 77% DNA sequences nearby the 376th codon were edited (Table 4).

**Table 3:** Results of the high-throughput sequencing of the sgRNA-ATG target site.

Wild type	5^′^-CATGCAGCCCGAACCCAAGC-3^′^
Editing	5^′^-CATGGCA--------GCCCGAACCCAAGC-3^′^ (43.82%)
	5^′^-CAT-----------------GCCCGAACCCAAGC-3^′^ (5.804%)
	5^′^-------------------------------------------AAGC-3^′^ (4.129%)

*sgRNA, single-guide RNA*.

**Table 4:** Results of the high-throughput sequencing of the sgRNA-376th target site.

Wild type	5^′^-CAACAACTCGCCGGACGGCA-3^′^
Editing	5^′^-C-AA-ACAA-C*---*TCGCCGGACGGCA-3^′^ (38.57%)
	5^′^-C-AA*--*CAA-CTCGTCGCCGGACGGCA-3^′^ (34.1%)
	5^′^-C*-----*CAA-C*---*TCGCCGGACGGCA-3^′^ (4.376%)

*sgRNA, single-guide RNA*.

We also performed western blot to detect the content of Keap1 on protein level. From the images of western blot, we observed that the ATG codon knock-out group has a lower Keap1 content than have the control group and the 376th codon knock-out group, whereas the protein content of the 376th codon knock-out group has no difference, compared with the control group (Figure S1).

**Figure 2:**
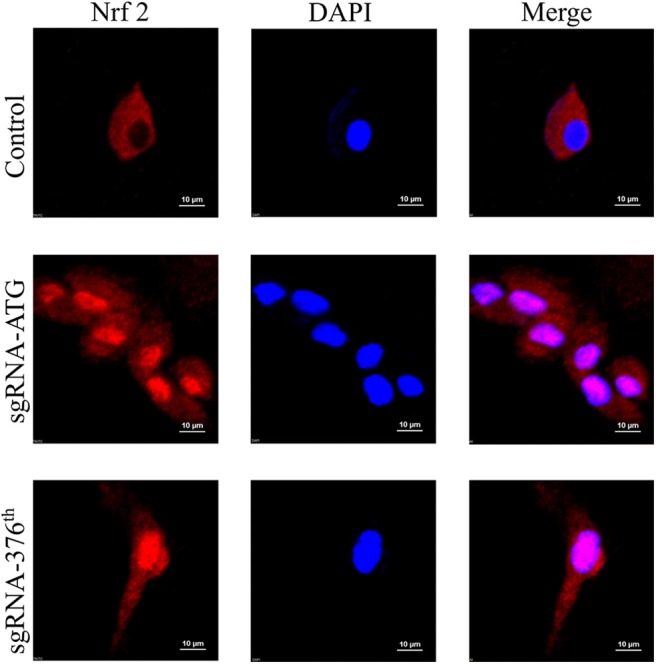
Images of immunofluorescence in each group represent localization of Nrf2; the pink surrounded by DAPI represents Nrf2 in nucleus.

After making sure that the recombinant CRISPR/Cas9 plasmid worked well in rat Ad-MSCs, we performed the experiment to determine whether Nrf2 was released and localized into nucleus. The immunofluorescence results indicated obvious nuclear localization in the Keap1 modified cells compared with the control group (Figure 2).

**Figure 3:**
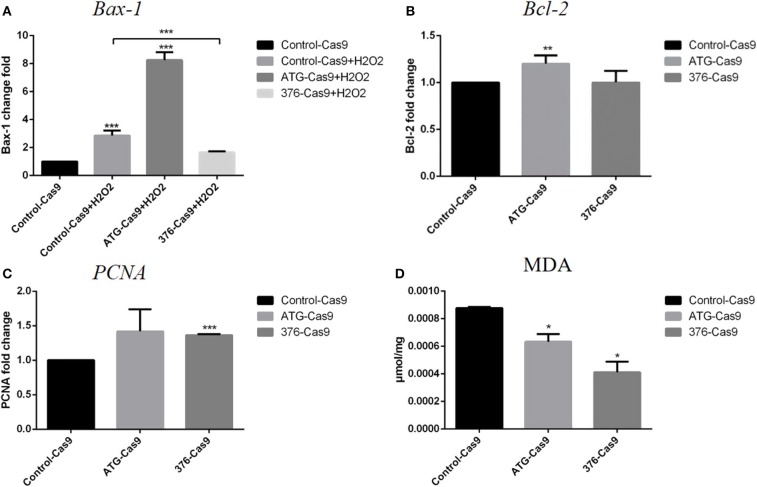
Evaluation of apoptosis and antioxidant abilities for the cells. Control-Cas9 represents the cells that were transfected with Cas9 only; +H_2_O_2_ means that the cells were treated with H_2_O_2_. The ATG-Cas9 represents the ATG codon of cells that were knocked out, and the 376-Cas9 represents the 376th codon of cells that were edited. **(A)** *Bax-1* expression of each group after being treated with H_2_O_2_. **(B)** *Bcl-2* expression of the control group and the gene-editing groups. **(C)** *PCNA* expression of the control group and the gene-editing groups. **(D)** Malondialdehyde (MDA) content of each group after H_2_O_2_ treatment. *N* = 3, *p ≤ 0.05, **p ≤ 0.01, and ***p ≤ 0.001.

We then detected the cell functions on growth, apoptosis, and their antioxidant ability in the modified Ad-MSCs and the wild-type control cells. The RT-qPCR was performed to detect the expression of a proliferation gene *PCNA*, an apoptotic gene *Bax-1* and anti-apoptotic gene *Bcl-2*. Ad-MSCs with a modified region at *Keap1* ATG codon showed a higher level of *Bcl-2* expression (Figure 3B), and meanwhile, the *Keap1* 376th codon-modified group displayed a higher level of *PCNA* expression, compared with the control cells (Figure 3C). Besides, we treated all groups of Ad-MSCs with 200 μM of H_2_O_2_ for 4 h and detected the expression of *Bax-1*. The result revealed that the group that was modified at the *Keap1* 376th codon showed a lower level of *Bax-1* expression, but the *Keap1* ATG codon mutation group possessed a higher expression of *Bax-1*, compared with the control cells (Figure 3A). MDA is one of the most important products of membrane lipid peroxidation, and its production can aggravate membrane damage. Therefore, MDA content is a common indicator in studies on oxidative stress. We treated all groups of Ad-MSCs with 200 μM of H_2_O_2_ for 4 h and detected the MDA content in each group. In both groups of the gene-edited Ad-MSCs, we observed a lower content of MDA, compared with the control group (Figure 3D).

## Discussion

Because of inflammation-induced blood blockage, and injury of astrocytes, which supply the nutrition, the energy need is blocked during traumatic brain injury, which might be the major reason for the cell death. Here, we explored the gene-editing technology using the CRISPR system to edit *Keap1* gene in mesenchymal stem cells so as to increase the anti-oxidative ability of the stem cells, helping them survive, and we found that the CRISPR/Cas9 gene-editing technology might be a good way to treat traumatic brain injury by enhancing cell anti-oxidant ability through gene editing.

The advent of the CRISPR/Cas9 gene-editing technology has revolutionized biomedicine, making it easier, faster, and cheaper to operate at the gene level. We try to apply it to stem cell therapy in nervous system disease through changing some characters of the stem cells. In previous studies, people used drugs to activate the Keap1–Nrf2 pathway, which is limited by the action duration of the drugs and failed to possess permanent reactions (17–19). And also in some studies, researchers precondition bone marrow mesenchymal stem cells to enhance their anti-oxidative ability (20). With the gene-editing strategy, we could modify gene sequences at specific regions of *Keap1* gene to promote anti-oxidant ability and most importantly obtain a permanent phenotype.

After introducing recombinant CRISPR/Cas9 plasmids into the cells using electrotransfection, we used FACS to screen out the positive cells by the mCherry gene in the plasmid, and then we used high-throughput sequencing to detect the proportion of DNA edited in the cells. The results showed that the efficiency of gene editing was relatively high (more than 50%). We then used immunofluorescence to detect that Nrf2 proteins were released into the nucleus, indicating that Nrf2 successfully localized into the nucleus. Subsequently, we used RT-qPCR to detect the expression of cell protection related genes, so as to prove that Nrf2 functions after entering the nucleus. The increased expression of *PCNA* gene was detected only in the 376th codon-modified group, and the only increase of *Bcl-2* appeared in the ATG codon-modified group, whereas the expression of *Bax-1* decreased significantly only in the 376th codon-modified group after the treatment with H_2_O_2_. We tested the antioxidant capacity of cells before and after gene editing, and we found that the gene-edited cells had significantly lower MDA content after oxidative stress than had the control cells, indicating that they had strong anti-oxidant capacity.

We noted some variations between those two types of gene editing from the experimental results. For example, there was no significant difference in the expression of proliferation gene *PCNA* between the *Keap1* gene ATG codon-modified group and the control group, and the expression of pro-apoptotic gene *Bax-1* was not decreased after treated with H_2_O_2_, but the expression of its anti-apoptotic gene *Bcl-2* was increased and the content of MDA was decreased. At the same time, in the treatment group where the sequences near the 376th codon were modified, the expression of pro-apoptotic gene *Bax-1* was decreased and the content of MDA was decreased as well, compared with the control cells under oxidative stress condition, representing significant enhancement of antioxidant capacity. However, the expression level of anti-apoptotic gene *Bcl-2* did not change significantly. Although the true reasons for these diversities have not been unrevealed in the present study, we assumed that the different regions in the genome of *Keap1* gene may play variable roles in anti-oxidation, and some unexpected off-targets that impact the related genes to result in the different expression of *Bax-1, Bcl-2*, and *PCNA* in the respective groups may have occurred. It deserves further experiments to confirm. In addition, we found that the Keap1 protein in each gene-editing group was still expressed at different levels (Figure S1), suggesting that these two gene-editing approaches just edited the key codon of domain but did not deplete the protein completely. We assume that this might be due to the limited efficiency of gene editing and that the total protein samples were from the mixture of edited cells and non-edited cells, because the primary stem cells cannot grow as single colonies. All the limitations of CRISPR/Cas9 in primary stem cells need to be improved in the future.

## Data Availability Statement

All datasets generated for this study are included in the article\Supplementary Material.

## Ethics Statement

This study was approved by the Ethics Committee for Animal Experiments of West China Hospital of Sichuan University, and all procedures were conducted in accordance with the guidelines of the NIH Animal Care and Use Committee.

## Author Contributions

YH and B-MZ designed the experiments and wrote the manuscript. YH performed the experiments and analyzed the raw data. SL performed the electrotransfection.

### Conflict of Interest

The authors declare that the research was conducted in the absence of any commercial or financial relationships that could be construed as a potential conflict of interest.
