# Supplementary material for: CRISPR/Cas9-Induced Loss of Keap1 Enhances Anti-oxidation in Rat Adipose-Derived Mesenchymal Stem Cells
Source: Front Neurol. 2020 Feb 18;10:1311. doi: 10.3389/fneur.2019.01311 (PMC7040357; doi:10.3389/fneur.2019.01311)
Supplement: Supplementary file 1 [file Image_1.pdf]

Supplementary Material

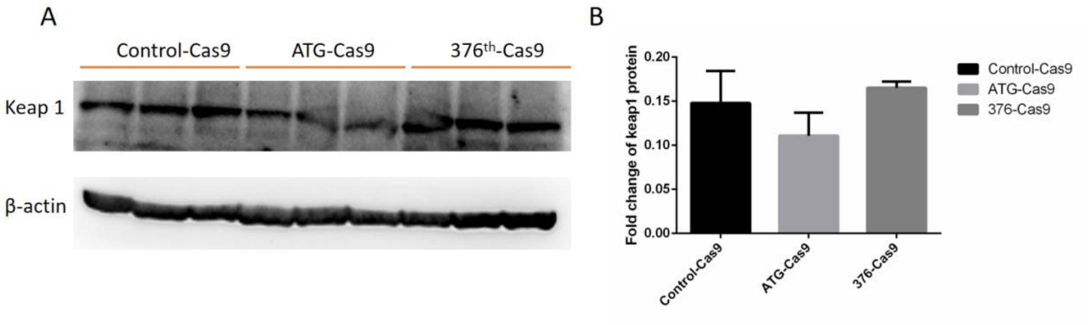

Figure S1. (A) Images of western blotting in each group represent keap 1 level after gene editing, (B) Semiquantitative analysis of keap1accumulation detected by Western blotting.
